# The Infection of Scarlet Fever

**Published:** 1896-10-10

**Authors:** J. O. Symes


### Medical Progress and Hospital Clinics.

[*The Editor will be glad to receive offers of co-operation and contributions from members of the profession. All letters should be addressed to* The Editor, at the Office, 28 & 29, Southampton Street, Strand, London, W.C.]

### THE INFECTION OF SCARLET FEVER.

J. O. Symes, M.D.Lond., D.P.H.

A great change has of recent years come over medical opinion as to the period of the disease during which the infection of scarlet fever is most virulent. The older authors held that the spread of infection was most to be feared during the period of desquamation, whilst more modern writers express the opinion that infection is less active about the end of the first week than it is either during the acute stage or after desquamation is well advanced. Goodall and Washbourne ("Manual of Infectious Diseases") still further modify these statements, and say, "Infectivity begins at the earliest stage of an attack, but is probably greatest when fever is highest." It is evident that the period of desquamation has come to be regarded as the period of least infectivity, and many medical practitioners have gone yet farther, and declared that the desquamation is not infectious at all, and in their management of cases act according to this belief.

Desquamation is not peculiar to scarlet fever. It follows attacks of measles, German measles, and erysipelas, and may often be detected after many other acute illnesses. In none of these is it regarded as the chief source of infection. It is, indeed, difficult to see that it should be so. Supposing that the cause of scarlet fever be a specific organism, the seat of inoculation is almost certainly the tonsil. Here, or in the blood, the poison is elaborated, and sets up that series of changes in the skin known as "the rash." It is hardly conceivable that the organisms themselves become first embedded in and finally cast off from the skin. Edington did, indeed, state that, after the third week, his bacillus was to be found in the desquamation; this, however, has not been confirmed. Klein's streptococcus scarlatinæ was detected in the blood, not in the desquamation, but this discovery, too, lacks confirmation.

But the question of the infectivity of the desquamative stage will finally be settled by the experience of every-day practice. Without doubt if, after the febrile state is over, the patient, whilst desquamating, be allowed to mix with susceptible persons, a certain proportion of these will contract scarlet fever. But such infection may be entirely independent of the desquamation. It may be the result of clothing imperfectly disinfected, of contamination from the breath, or of infectious discharges from nose or ears. Desquamation alone would seem in some cases at least to be insufficient. For instance Dr. Priestly, of Leicester, had, in consequence of an outbreak of small-pox in the town, to clear the infectious diseases hospital. (See "Proceedings Epidem. Society, 1885.") He sent home 120 cases of scarlet fever in various stages of desquamation, and yet during the following three months not a single second case occurred in any one of their houses. On a second occasion he sent home twenty-three cases in whom desquamation was incomplete, and again there were no "return cases."

Dr. Gilbert, of Tunbridge Wells, writing to the *British Medical Journal*, September 14th, 1895, stated that he isolated his patients for a month only, and then, whilst still desquamating, allowed them to mix with others who had never had the disease. No ill effects ensued. It is true that during the period of isolation their skin had been treated with carbolic oil, but no one acquainted with the difficulties met with in the attempt to sterilize the skin can for a moment regard this treatment as sufficient to secure disinfection. Neither can we imagine that Tucker's eucalyptus disinfection fluid (a mixture of eucalyptus, with menthol and essential oils and camphors) is sufficiently potent to disinfect the surface of the body; yet Dr. Priestly and Dr. Curgenven have treated a large number of cases by inunction with this preparation, allowing them to mix with their friends after the tenth day of the disease, and no ill effects have resulted. All these facts point to the lessened infectivity of the peeling stage rather than to successful disinfection of the skin. Undoubtedly cases of scarlet fever may be sources of infection weeks after the commencement of the attack. The average stay of a patient in the hospitals of the Metropolitan Asylums Board is seventy days, and in the Leicester Hospital thirty-seven days only. As far as one can judge, the peeling of the Leicester patients must be much less perfect than that of the London patients, but there is no corresponding increase in the number of "return cases." The report of the Metropolitan Asylums Board stated that one case in 285 gave rise to a "return case," and the question naturally arises, If the peeling be not the cause, what is?

The occurrence of a second case of scarlet fever in a household to which a convalescent case has recently returned may be due to one of several causes. Many attacks occurring fourteen or more days after the return of the convalescent may be regarded as instances of infection contracted from some other source. Fully 5 per cent. of the cases may be thus explained. Others undoubtedly arise owing to the disturbance of insufficiently disinfected clothes, which are left at home, stored away during the patient's stay in hospital, only to be produced on their return. As soon as efficient steam-disinfecting apparatus is more widely used, the number of such accidents will diminish. By far the greater number of return cases arise, however, from the fact that patients are discharged before the secondary inflammations and discharges are cured. Dr. Boobyer, of the Nottingham Borough Isolation Hospital, examined twenty-nine discharged patients, who had, on their return to their homes, been the means of spreading infection. All were sent out apparently well. Their stay in hospital had averaged 8·1 weeks, the longest being twelve and the shortest being six weeks. At the time of examination none were peeling, but five had inflamed throats, seven discharge from the nose, three discharge from the ear, and three nephritis, whilst the remaining eleven were apparently quite well. All these gave rise to home cases, the shortest interval between the return of the primary case and the development of symptoms in the secondary case was twenty-four hours, the longest twenty-nine days. Similarly of five cases reported by Dr. Priestly as giving rise to return cases, two had suppuration from the middle ear; and of eleven reported by Dr. Cooper-Tattin (M.O.H., Norwich), five had rhinorrhœa. It were easy to multiply like instances, but sufficient has been said to show the danger that may arise by discharging patients before all secondary discharges have ceased But it is not merely a matter of secondary discharges. What is required is a thorough disinfection of the whole naso-pharynx. The patient has been breathing an atmosphere laden with the poison of scarlet fever for weeks, and no amount of gargling, spraying, irrigation, or inhalation will free the respiratory tract from infection. The breath of a child recently discharged from a fever ward is a fruitful source of infection. Dr. Chalmers (M.O.H., Glasgow) investigated a series of 118 return cases. Of these, 93 per cent. occurred within fourteen days of the discharge of the primary case, and for the most part the secondary case was sleeping in the same bed as the first sufferer. By making a comparison of the two city hospitals, Dr. Chalmers pointed out that overcrowding played an important part. The air of the crowded wards was more deeply polluted, the patients were discharged in a more highly infective state, and consequently an overcrowded hospital led to an increase in the number of return cases. It is not uncommon when inquiring into the cause of a return case to find that the primary case has peeled a second time after convalescence had been pronounced complete; and here it might be thought that certainly the desquamation were the infective agent. Without denying that this may be so, it is well to remember that the second desquamation may be but a consequence of a renewed activity of the disease, and that the true contagium may be the exhalations of the patient, more particularly the breath.

Reviewing the whole of the evidence before us, we cannot fail to regard the infective property of the desquamation in scarlet fever as of secondary importance, and to attach more weight to the necessity of preventing the spread of infection by secondary discharges, or by the exhalations. In this way we shall come to class the infective properties of scarlet fever with those of erysipelas and diphtheria, to which diseases it is, indeed, closely allied. When the bacteriology of scarlet fever is fully known, then, and not until then, shall we be able definitely to decide whether the desquamation is or is not infectious. In the meantime, the safest method to pursue is to allow the full period of six weeks' isolation, and to permit a further fortnight to elapse before the patient rejoins the family circle. The greater part of this last period should be spent in the open air, for in this way only can the respiratory tract be rendered free from infection. Fresh air and sunshine are here, again, the only effective purifiers. In no case should the patient be allowed to return home whilst there is a secondary discharge of any nature. With these precautions return cases seldom or never occur.

